# Evaluation of Antibacterial Mechanism of Action, Tyrosinase Inhibition, and Photocatalytic Degradation Potential of Sericin-Based Gold Nanoparticles

**DOI:** 10.3390/ijms24119477

**Published:** 2023-05-30

**Authors:** Gitishree Das, Jayanta Kumar Patra

**Affiliations:** Research Institute of Integrative Life Sciences, Dongguk University-Seoul, Goyangsi 10326, Republic of Korea; gdas@dongguk.edu

**Keywords:** gold nanoparticles, sericin, antibacterial activity, mechanism of action, tyrosinase inhibition, photocatalytic degradation

## Abstract

In recent times, numerous natural materials have been used for the fabrication of gold nanoparticles (AuNPs). Natural resources used for the synthesis of AuNPs are more environment friendly than chemical resources. Sericin is a silk protein that is discarded during the degumming process for obtaining silk. The current research used sericin silk protein waste materials as the reducing agent for the manufacture of gold nanoparticles (SGNPs) by a one-pot green synthesis method. Further, the antibacterial effect and antibacterial mechanism of action, tyrosinase inhibition, and photocatalytic degradation potential of these SGNPs were evaluated. The SGNPs displayed positive antibacterial activity (8.45–9.58 mm zone of inhibition at 50 μg/disc) against all six tested foodborne pathogenic bacteria, namely, *Enterococcus feacium* DB01, *Staphylococcus aureus* ATCC 13565, *Listeria monocytogenes* ATCC 33090, *Escherichia coli* O157:H7 ATCC 23514, *Aeromonas hydrophila* ATCC 7966, and *Pseudomonas aeruginosa* ATCC 27583. The SGNPs also exhibited promising tyrosinase inhibition potential, with 32.83% inhibition at 100 μg/mL concentration as compared to 52.4% by Kojic acid, taken as a reference standard compound. The SGNPs also displayed significant photocatalytic degradation effects, with 44.87% methylene blue dye degradation after 5 h of incubation. Moreover, the antibacterial mode of action of the SGNPs was also investigated against *E. coli* and *E. feacium*, and the results show that due to the small size of the nanomaterials, they could have adhered to the surface of the bacterial pathogens, and could have released more ions and dispersed in the bacterial cell wall surrounding environment, thereby disrupting the cell membrane and ROS production, and subsequently penetrating the bacterial cells, resulting in lysis or damage to the cell by the process of structural damage to the membrane, oxidative stress, and damage to the DNA and bacterial proteins. The overall outcome of the current investigation concludes the positive effects of the obtained SGNPs and their prospective applications as a natural antibacterial agent in cosmetics, environmental, and foodstuff industries, and for the management of environmental contagion.

## 1. Introduction

Presently, bacteria in different forms are resistant to existing antibiotics, and the number of antibiotic resistant organisms is rising progressively, which is a severe risk to public health in numerous parts of the world, affecting both developed and developing nations [1,2]. The cause of the occurrence of many infectious diseases, such as chronic obstructive pulmonary disease, pneumonia, trichomoniasis, etc., and subsequent deaths due to these diseases, are usually associated with pathogenic microorganisms [3]. For the treatment of these ailments, often the available antibiotics are preferred, as these antibiotics control the growth of harmful microbes, and thus reduce the possible side effects [3]. However, over the last few decades, the appearance of multidrug-resistant pathogens and the sustainable application of available antibacterial and antifungal drugs have been a major cause of concern [4,5,6]. Antibiotics are often ineffective at handling diseases initiated via drug-resistant bacteria. These issues have given rise to the demand for the development of novel drugs and drug candidates for the treatment of bacteria-, virus-, and fungal-infection-related diseases. A stronger substitute solution is needed to enhance the effects of these resistant antibiotics against pathogenic microorganisms [1,7]. Instead of antibiotics for treatment, it is necessary to identify novel antibacterial agents [8,9,10,11].

In the last two decades, the study of nanotechnology has developed as an encouraging field of interdisciplinary study because of its enormous application across a varied field of scientific research [12]. Presently, metallic nanoparticles have been used in various applications, including medicines, the food sector, agricultural products, and cosmetics [12,13]. However, the procedures for the industrial-scale manufacturing of nanomaterials are not environmentally friendly, and they usually follow the physical and chemical methods of synthesis that involve the utilization of toxic chemicals, which is not only hazardous to the environment, but also gives rise to numerous toxic byproducts [12]. To counter this process, the green technology or biological-based synthesis procedure is an effective alternative tool for the manufacture of non-toxic and environmentally friendly materials [14,15]. Nanomaterials produced by biological processes are diverse in nature, with more stability, and above all, are non-toxic and environmentally friendly [15].

Nanoparticles are extensively used, and play a major role in a variety of fields, including biology, medicine, physics, chemistry, and sensing, due to their exclusive potential [16,17]. Many researchers have unveiled that nanoparticles with various properties, such as small size, distinct shape, high surface charge and area, capacity to carry a large number of antibacterial compounds, etc., are a perfect antimicrobial weapon that can prevent the spread of harmful microbes and be used to explore their pathogenicity [3,18,19]. Nanoparticles possess antimicrobial potentials that can overcome common disease-resistant mechanisms, such as the modification of enzyme inactivation, cell permeability, active efflux, and drug targeting, to escape from the antimicrobial agent’s antibacterial effect [1,20,21]. The use of nanoparticles offers a prospective approach to control contagions initiated by multidrug-resistant bacteria [1]. Antibiotics may be substituted with metal-based nanoparticles that have enduring antibacterial effects [7]. Some of the basic modes of action of the nanoparticles, as reported previously, include cell wall penetration, cell membrane damage, and loss of membrane protein and deoxyribonucleic acid (DNA), which cause the inhibition of bacteria and other microorganisms [3].

Among the nanoparticles, gold nanoparticles (AuNPs) are one of the most highly important nanoparticles, and have attracted much interest from researchers due to their unique properties, such as small size, ionic neutrality, and precise targeting [22,23,24,25]. The variety of AuNPs used in modern medical and biological studies is extensive [26]. They have a wide variety of applications in therapeutics, diagnostics, drug and gene delivery, catalysis, electronics, chemical and biological imaging, biomedical arenas, optics, etc. [9,10,11]. They have been used in drug delivery systems for therapeutic agents, in cancer treatment, and also as a diagnostic tool in the detection of several biomarkers for different diseases [27]. AuNPs have been extensively used in the identification of chemical and biological agents [26]. The utilization of AuNPs in biomedical imaging techniques, such as photo-acoustic imaging, X-ray computed tomography, dark-field microscopic imaging, fluorescence imaging, and magnetic imaging, is very popular [23,24,28,29]. It has been claimed that AuNPs can be used in nearly all medical applications, such as disease diagnostics, treatment, prevention, and also in the maintenance of hygiene [26]. It has been reported that the antibacterial action of the gold nanoparticles is probably due to their small size: they accumulate at a specific location of the bacterial cells, resulting in the rupture of the membrane and cellular lysis [9,30]. It was also clear from the small-angle X-ray scattering analysis of earlier reports that the AuNP conjugates adhere to and penetrate the cell wall of bacteria, resulting in the disruption of the cellular environment, which leads to cell lysis owing to the leakage of cellular components [31].

In the current times, the environmental pollution caused by the textile, paper, food, and cosmetics industry dyes such as methylene blue and methyl orange is a major concern throughout the world [32,33]. These dyes not only affect the environment, but also aquatic organisms and human beings, due to their carcinogenic and toxic properties [32,34,35]. It is always difficult to remove the dyes from the environment due to their complicated chemical nature [32,36,37]. In recent times, with the advancement in the field of nanotechnology, numerous nanomaterials have been used in the dye degradation process because of their distinct properties, such as small size, high surface area, recyclability, etc. [38,39]. Further, it is known that light has a surface plasmon resonance effect on the nanoparticles, and thus, it can be considered a promising candidate to act as a photocatalytic agent for the degradation of various industrial dyes [40].

Sericin is a globular protein, usually discarded in the process of manufacturing silk from the silk cocoon by the sericulture and textile industries [41,42,43]. Sericin has been reported to possess 18 amino acids with polar side chains comprising amino, hydroxyl, and carboxyl groups [42,43]. These distinct properties of sericin enable easy copolymerization, cross-linking, and blending with other compounds to form complex materials with superior properties [43,44]. These above-mentioned potentials of sericin proteins were accredited to the higher quantity of polar amino acids with hydroxyl groups (such as serine and threonine), which are present in abundant quantities within the secondary structure of the silk sericin [43]. Previous reports have proved the potential applications of sericin and sericin-based materials in terms of their antimicrobial, wound healing, antitumor, anticancer activities, etc. [41,42,43,44,45,46,47]. In light of this potential of sericin, in the existing analysis, the antibacterial effect, antibacterial mechanism of action, tyrosinase inhibitory effect, and photocatalytic degradation effects of sericin-fabricated gold nanoparticles (SGNPs) were explored.

## 2. Results and Discussion

### 2.1. Assessment of Antibacterial Effect and Action Mechanism of SGNPs

The antibacterial effectiveness of SGNPs was estimated for six (three Gram-positive and three Gram-negative) bacterial pathogens, namely, *E. feacium*, *S. aureus*, *L. monocytogenes*, *E. coli*, *P. aeruginosa*, and *A. hydrophila*, and the results are displayed in Table 1. The inhibition results ranged between 8.45 mm to 9.58 mm (Table 1). The results disclose that the SGNPs were harmful to all the bacterial pathogens at the lowest tested concentration, 50 μg/disc. The positive control was taken as Cephalexin (10 μg/disc): it displayed a zone of inhibition that ranged between 12.14–12.23 mm (Table 1). Five % DMSO, taken as the negative control, did not show any inhibition activity against the tested microbes. The MIC and MBC were also determined, and are presented in Table 1. Some of the results in terms of MIC and MBC have already been studied previously by our group [41]. Similar results on the antibacterial activity of the AuNPs were presented in the previously published literature [48], where it is shown that the growth of the *E. coli* and *P. aeruginosa* pathogens completely ceases at the concentrations of 100 μg/mL and 150 μg/mL, respectively. Further, in another study, De Lima Oliveira et al. [49] discussed the synergistic antibacterial effect of the AuNPs against *S. aureus*, and found that the MBC value was 12.5 mg/mL. In a different study, the authors discussed the antibacterial effect of sericin against many pathogenic bacteria, namely, *Bacillus subtilis*, *Staphylococcus aureus*, and *Staphylococcus epidermidis* [43]. Since the current SGNPs were prepared using sericin protein as the reducing and capping agent, it could have been helpful in the exhibition of the positive antibacterial potential of the material.

### 2.2. Assessment of Antibacterial Mechanism of Action

**Figure 1:**
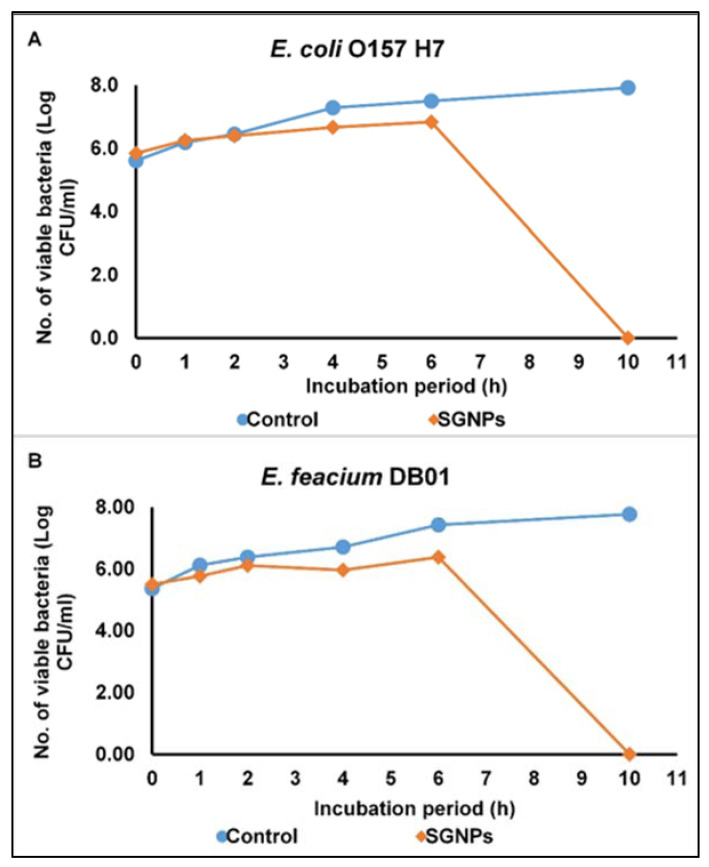
Effect of SGNPs on the viability of the bacterial cell counts. (**A**) *E. coli* and (**B**) *E. feacium*.

The antibacterial mode of action effect of SGNPs was evaluated by using two selected pathogens (*E. coli* and *E. feacium*). The outcome is displayed in Figure 1. The SGNPs at the MIC did not display a considerable decline in the viability count of either of the microbes up to 6 h, but it significantly declined at 10 h (Figure 1A,B). For the standard (control), the progress of the pathogen growth constantly rose with the time of incubation. It could be inferred that the SGNPs, because of their nanoscale size, could have easily invaded the cells of the bacteria, causing widespread destruction of the membrane wall, and eventually ending in cellular demise [50,51].

The AuNP antimicrobial action for Gram-positive and Gram-negative bacteria is diverse, and the structure of the membrane is the key difference [8]. The consistency of the peptidoglycan layer is a vital portion of the infective bacteria. It is 50% greater in Gram-positive microbes than in Gram-negative. So, usually, in the case of the Gram-positive bacteria, higher doses of NPs are needed [8,52]. However, in the current investigation, it seems a similar dose the SGNPs is required for both tested pathogens. The nanoparticles upset the usual functions of the cellular proteins, which leads to cell death [8].

**Figure 2:**
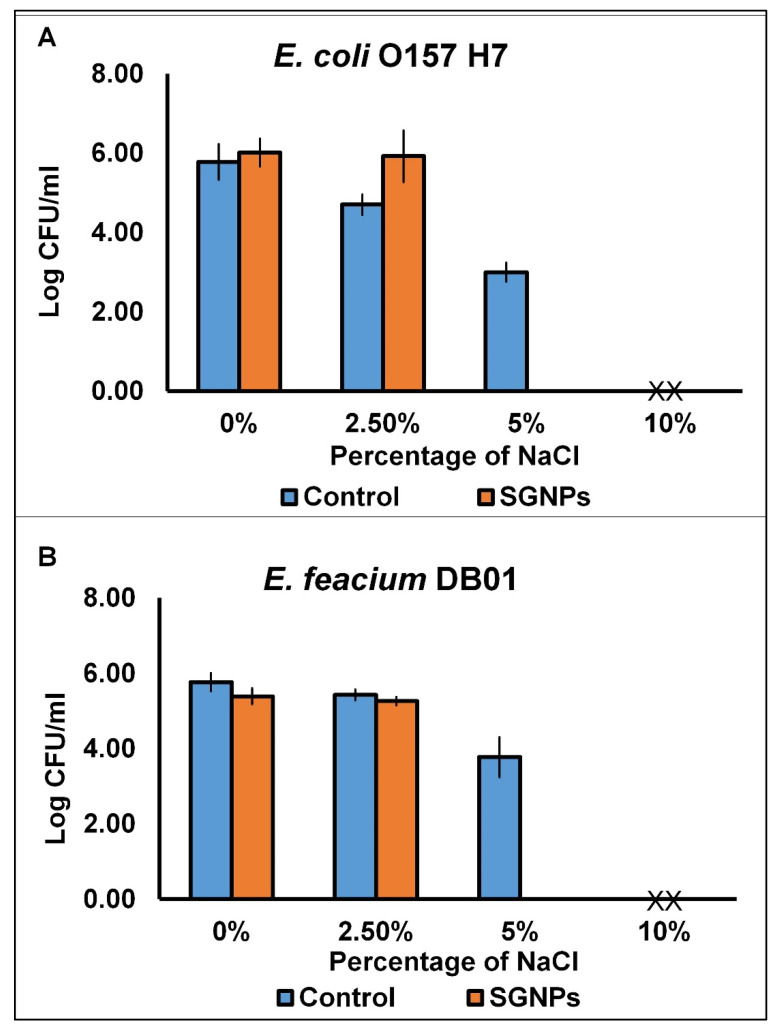
Effect of SGNPs on salt tolerance. (**A**) *E. coli* and (**B**) *E. feacium*.

For the SGNP salt tolerance analysis, the nutrient agar plates inoculated with SGNPs were supplemented with different concentrations (0–10%) of sodium chloride. When the NA plates of the SGNP-treated microbes were observed, it was found that at 0% and 2.5% salt content, there is much less of a decline in the bacterial count (cfu) (Figure 2). However, the development of both the bacteria pretreated with SGNPs was terminated at 5% salt concentrations, whereas in the case of unprocessed control microbes, the growth was sustained until 5% salt content (Figure 2). Hence, the salt forbearance function of the tested microbes, which were pretreated with the SGNPs (at MIC concentrations), is another factor that supports the claim of bacterial cell membrane damage, owing to the assembly of SGNPs on the microbial cell surface [53]. Injury caused by the treatment of toxic materials on the bacterial cell membranes might modify the cell membrane’s capability to osmoregulate the cell effectively, or to eliminate toxic materials from the body [54,55,56]. Subsequently, the loss of capacity to tolerate different concentrations of salts or any toxic material can be explored to determine the membrane damage of the injured microbes [54,57]. Treatment of tested microbes with SGNPs at MIC concentration expressly decreased the ability of the surviving bacteria to form colonies on the nutrient agar plates containing high concentrations of sodium chloride salt; these results corroborate the findings of the next experiment on the effect of SGNPs on the release of 260 nm absorbing materials.

**Figure 3:**
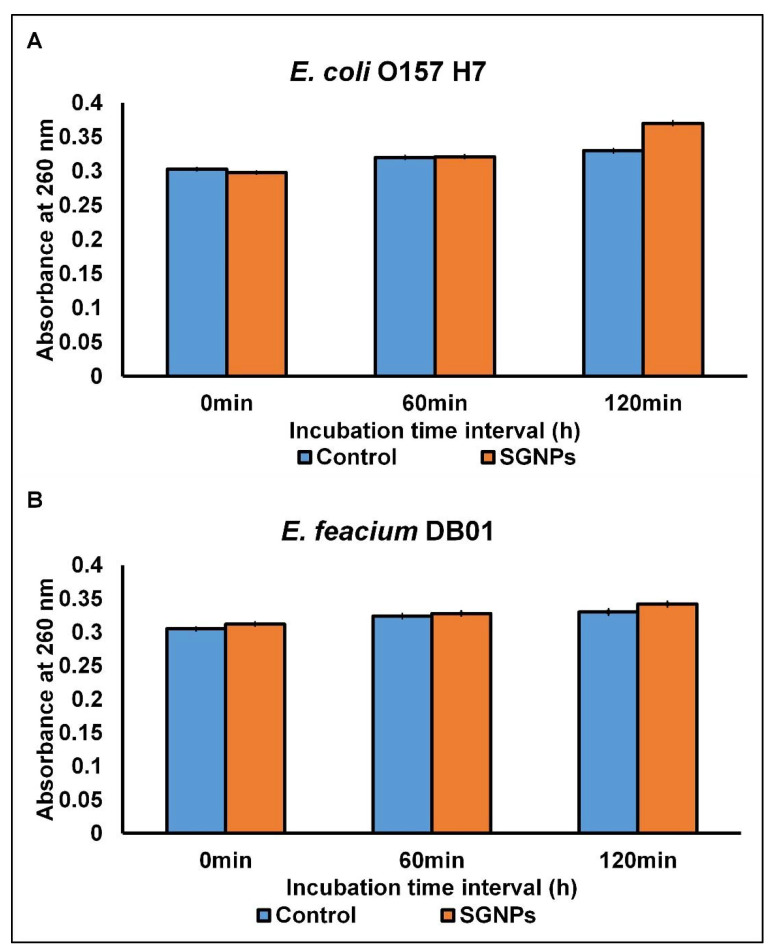
Effect of SGNPs on the release of 260 nm absorbing constituents. (**A**) *E. coli* and (**B**) *E. feacium*.

For the discharge cellular materials (detected at 260 nm), when both *E. coli* and *E. feacium* (MIC concentrations) were treated with SGNPs, there was a persistent rise in the concentration of cellular materials absorbed at 260 nm in a spectrophotometer during the period of incubation (Figure 3). However, in the control without SGNPs treatment, the concentration of cellular materials remains persistent, and after an incubation time of 1 h, there is no change in the OD values (Figure 3). Specifically in *E. coli*, a significant upsurge in the OD value at 260 nm specifies a strong release of cellular contents (such as proteins and nucleic acids) to the outer atmosphere because of cell membrane leakage [56]. Noticeable leakage of the cytoplasmic membrane materials is considered an indication of mass and irreparable injury to the cytoplasmic membrane of the microbes [58]. Several antimicrobial compounds that acted on the bacterial cytoplasmic membrane have been reported to have induced the loss of 260 nm absorbing materials [54]. The above results conclude that the mode of antimicrobial potential of SGNPs might be owing to their relatively small sizes and their high capability to attach to the exterior microbial wall, and ultimately, they could possibly enter the cell and instigate cell membrane waning, which could possibly result in the seepage and injury of cellular constituents, leading to cellular lysis [58]. These above results relate well with the 260 nm release results since, in each case, treatment with SGNPs at the MICs prompted the loss of salt tolerance and 260 nm absorbing materials in both the tested bacteria.

**Figure 4:**
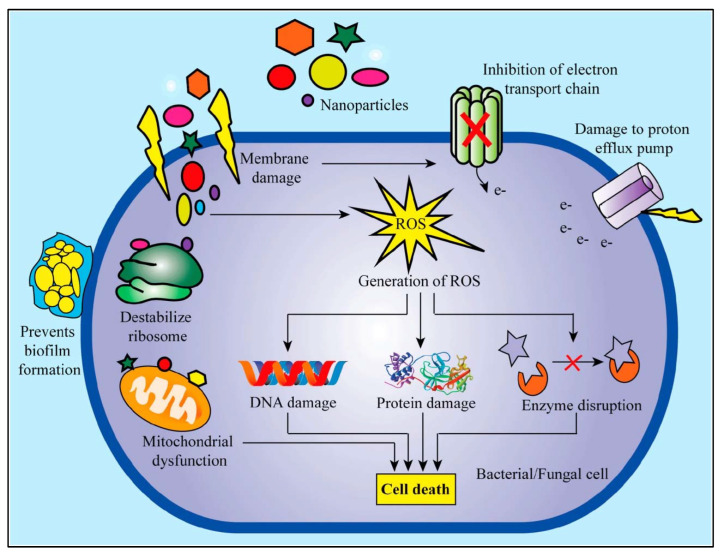
Pictorial representation of the mode of antimicrobial action of nanoparticles. Reproduced with permission from Sharmin et al. [3], (originally Figure 7).

Some reports show that the possible mode of antimicrobial action by nanoparticles could occur in several ways, such as inducing oxidative stress in the bacterial cells, releasing the metal ions into the cell membrane, and other non-oxidative actions, and it has been stated that due to the small size of the nanomaterials, they could have adhered to the surface of the pathogens, and could have released more ions and dispersed in the bacterial cell wall surrounding environment, thereby disrupting the cell membrane and ROS production, and subsequently penetrating the bacterial cells, resulting in lysis or damage to the cell by the process of structural damage to the membrane, oxidative stress, and damage to the DNA and bacterial proteins [2,3,59]. In a previous study, the authors reported that the gold nanoparticles induce vesicle formation, resulting in the rupture of the cell membrane in the case of the *E. coli* pathogen [60]; in another study, the nanoparticles facilitated the increase in the concentration of the reactive oxygen species, ultimately leading to cell death [61]. These findings are also proven in the current investigation (Figure 1, Figure 2 and Figure 3). All the possible mechanisms of action of the nanoparticles are explained in pictorial form in Figure 4.

Overall, the possible mode of antibacterial action of the SGNPs can be explained in many different ways. It is usually seen that when the metal ions dissolved in solvents come into contact with a bacterial cell, they become mostly detached from the surrounding environment of the bacterial cell [9]. However, some of the NPs that were attached to the bacterial cell wall at a specific location induced cell toxicity by the continuous release of ions, and thereby lysis of the bacterial cells [62]. In the current investigation, it can be seen that the SGNPs have a slightly better effect against the Gram-negative bacteria (Table 1), which could be possibly due to the thin cell wall of the Gram-negative bacteria, which facilitates easy penetration of the SGNPs into the bacterial cell membrane, causing cellular damage [9,63,64]. Many previously published reports suggested that, usually, the antibacterial activities of any drug are accomplished by two types of mechanisms, first by the inhibition of metabolic processes by altering the membrane potential and decreasing the ATP synthase activity, and secondly, by rejecting the ribosome’s subunit for tRNA binding by efficiently disassembling the whole bacterial process [8,9]. There are reports that gold NPs are the most common and widely studied metals for antibacterial action due to their enhanced property of electronic effects and high surface area percentages, which are responsible for enhanced antibacterial action [65,66]. The synthesized SGNPs could be possibly verified for their uses in the biomedical, food, and cosmetics trades.

### 2.3. Assessment of Tyrosinase Inhibition Effect of SGNPs

**Figure 5:**
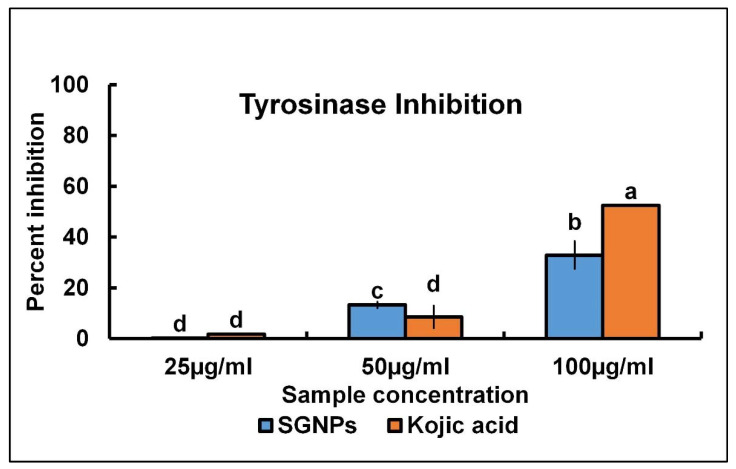
Tyrosinase inhibition potential of SGNPs and standard Kojic acid. The differences in the superscript letters at each bar are statistically significant at *p* < 0.05.

The results of tyrosinase enzyme inhibition are displayed in Figure 5. The SGNPs showed a moderate tyrosinase inhibition effect with 32.83% inhibition (at 100 μg/mL). Meanwhile, the Kojic acid (reference standard) exhibited 52% inhibition at the same concentration (100 μg/mL) (Figure 5). However, at 50 μg/mL concentration, SGNPs displayed an inhibition effect with 13.26% inhibition, whereas Kojic acid exhibited 8.52% inhibition (Figure 4). Similar positive results on the tyrosinase inhibition effects of gold nanoparticles were also reported previously [67].

Melanin is a factor in skin coloration, and is produced by the melanogenesis pathway in the melanocyte cells situated in the lower epidermis [68]. Melanin is formed by L-tyrosine oxidation, followed by transformation to L-dihydroxyphenylalanine [50,69]. Despite its defensive functions, such as protecting the skin from the adverse effect of harmful ultraviolet rays, its surplus formation resulted in the production of age spots, freckles, hyperpigmentation, and melisma [70,71,72,73]. The tyrosinase inhibition activity by various medications is an effective method of treatment for skin-related ailments [74]. There are reports that sericin protein has potential tyrosinase inhibition effects [75,76,77]. In a previous study, Cherdchom et al. [75] reported on the anti-melanogenic potential of urea-extracted sericin protein by the process of tyrosinase inhibition activity by reducing the formation of B16F10 cells without the induction of cytotoxicity. The SGNPs used in the current study were prepared by using the sericin protein as a reducing agent. Further, sericin protein also acted as the capping and stabilizing agent for the nanoparticle. Hence, the medicinal effects of sericin were also added to the SGNPs, and the tyrosinase effect of the SGNPs is a collective effect of both.

There is remarkable interest among the cosmetic industries in the formulation of natural products with anti-tyrosinase activity for the manufacture of a low-cost, herbal, and highly effective anti-tyrosinase product for bleaching off exceedingly pigmented lesions [78]. Therefore, the usage of SGNPs with effective anti-tyrosinase potentials could be potentially utilized by cosmetic companies after detailed assessments and endorsements.

### 2.4. Assessment of Photocatalytic Degradation Effect of SGNPs

**Figure 6:**
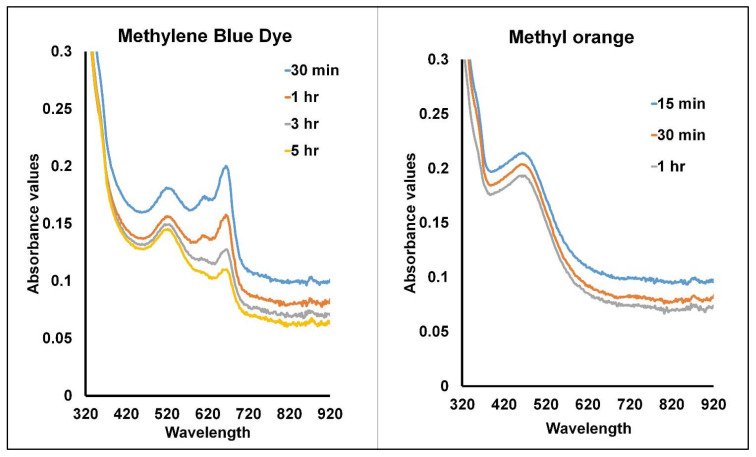
Photocatalytic degradation of methylene blue and methyl orange dyes by SGNPs.

The SGNPs facilitated the photocatalytic degradation of dyes (methylene blue and methyl orange) with light exposure. The results depict that with an increase in the light exposure time, the OD values declined for MB in the presence of SGNPs (Figure 6). At diverse time intermissions until 5 h, the effectiveness of the SGNPs for the degradation of MB was assessed at 668 nm. After adding SGNPs to the MB solution with light treatment, a time-dependent decline in the OD value was observed (Figure 6). The calculated MB degradation percentage was around 44.87% (Figure 6). Similarly, in the case of the MO, the absorbance band for MO also dropped with an increase in the light exposure period (Figure 6). Until 60 min at diverse time intervals, the efficacy of the SGNP-mediated MO degradation was recorded at 464 nm. When SGNPs were added to the MO solution and exposed to light treatment, they exhibited a time-dependent drop in the absorption peak value intensity. After light exposure for 60 min, it was observed that the percentage of methyl orange dye degradation was about 9.79% (Figure 6).

The current results specify that the cationic dyes (methylene blue and methyl orange) demonstrated a decrease in the absorbance with the increase in the incubation time; this suggests the selective interaction of the cationic dyes with the SGNPs that facilitates their degradation in the reaction solution. As reported by Singh et al., the elimination of dyes from the solution is affected by several aspects, such as electrostatic interaction, pore filling, or hydrogen bonding, and the variation of the surface charge is influenced by the pH of the solution [32]. Thus, in the current study, since the SGNPs are synthesized by the sericin protein, and the solution is alkaline, with a pH of more than 7, they facilitated the declination of MB, as evident from the results. It could also be stated that the phenolic compounds of sericin, represented as the covering agent in SGNP biosynthesis, might play a momentous part in the adsorption of MB and MO, representing the likelihood of the electrostatic relations between the dye molecules and the phenolate [32,79].

## 3. Materials and Methods

### 3.1. Resources

**Figure 7:**
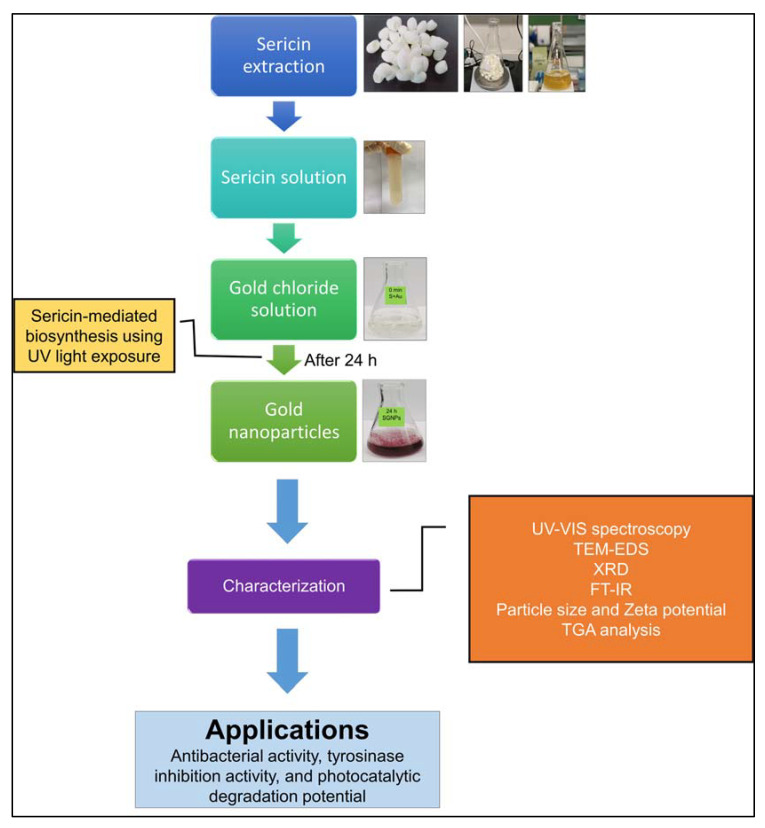
Schematic diagram of a procedure of sericin-mediated biosynthesis of SGNPs.

The extraction, purification, and synthesis of sericin-based gold nanoparticles (SGNPs), and synthesized SGNP characterization is detailed in our earlier published research [41]. A schematic diagram for the biosynthesis procedure of SGNPs is shown in Figure 7. The antibacterial effect study of SGNPs was performed against pathogenic bacteria, including *Enterococcus feacium* DB01, *Staphylococcus aureus* ATCC 13565, *Listeria monocytogenes* ATCC 33090, *Escherichia coli* O157:H7 ATCC 23514, *Aeromonas hydrophila* ATCC 7966, and *Pseudomonas aeruginosa* ATCC 27583.

### 3.2. Determination of In Vitro Antibacterial Efficacy

The antibacterial effect of SGNPs was assessed against both Gram-positive and Gram-negative pathogenic bacteria, as earlier described [80]. The bacteria were subcultured in a culture medium before use, and before the experiment, the bacterial cultures were adjusted to 1.5 × 10^8^ cfu/mL (0.5 McFarland standard). The disc diffusion assay was used for the antibacterial assay. A paper disc of 50 μg SGNPs/disc was prepared for the disc diffusion assay. On the prepared agar plates, the freshly subcultured pathogens were spread evenly. Next, the filter paper disc with SGNPs was placed onto the agar plates and stored for 24 h at 37 °C for incubation. After the incubation time, the inhibition consequence of SGNPs was measured by the zone of inhibition diameter. For the antibacterial assay, we have used the standard antibiotic cephalexin as the positive control and the 5% DMOS as the negative control. The minimum inhibitory (MIC) and minimum bactericidal concentrations (MBC) were also determined as per previously standardized two-fold serial dilution and plate count methods [41].

### 3.3. Determination of the Antibacterial Mode of Action

#### 3.3.1. Cell Viability Effect

The antibacterial mode of action of SGNPs on the tested Gram-positive and Gram-negative microbes was determined following the time–kill assay, as discussed in our previous study [50]. For the antibacterial mode of action studies, only two pathogens, *E. coli* and *E. feacium*, were selected from among the six tested pathogens due to the better inhibition activity of the SGNPs. Initially, the pathogenic bacterial culture was used at the MIC concentration. The bacterial culture without any treatment was taken as the control. The culture solution was 10 mL (10^7^ cfu/mL), and the nanoparticles were kept for 10 h at 37 °C for incubation. The bacterial colony was counted as cfu/mL.

#### 3.3.2. Assessment of Salt Tolerance Capacity

The salt tolerance potential of the investigated microbes, used to find out the capability of the microbes to osmoregulate, was investigated by following the standard procedure [50]. The pathogenic microbes after SGNP treatment (MIC concentration) were incubated at 37 °C for 1 h. Next, they were sowed onto the agar dishes after serial dilution comprising salt with different concentrations (0–10.0%). As the positive control, the pathogenic bacteria culture without treatment of SGNPs was taken. The number of microbial colonies was recorded as Log10 cfu/mL.

#### 3.3.3. Assessment of the Bacterial Effect on Cellular Materials Absorbing at 260 nm

The release of cellular materials from the microbial cells was determined using standard procedures [50]. Before the experiment, the pathogens were treated with SGNPs (at MIC concentration), added to 2.0 mL microbial culture, and incubated (at 37 °C). Next, the microbial solution was eluted and centrifuged (at 1.5 × 10^3^ rpm for 10 min), followed by the recording of the optical density (OD) at 260 nm at constant time intervals. In this experiment, peptone water mixed with microbes and SGNPs, separately, was taken as the negative and positive control, respectively.

### 3.4. Assessment of Tyrosinase Inhibition

The evaluation of the tyrosinase inhibition effect of SGNPs was conducted by a spectrophotometric technique [74]. Concisely, the reaction mixture solution (300 μL) contains 25–100 μg/mL of SGNPs, PBS (0.1 mM, pH 6.5), L-DOPA 0.1 mM, and mushroom tyrosinase 50 U/mL. The sample was kept for 1/2 h at ambient temperature, then the OD was detailed at 475 nm. The positive control used was Kojic acid. The tyrosinase inhibition percentage is formulated below:

$$\mathrm{Percentage}\;\mathrm{inhibition}=\frac{C_{s}-T_{s}}{C_{s}}\times 100$$

“*C_s_*” is the OD of control and “*T_s_*” is the OD of the test.

### 3.5. Assessment of the Photocatalytic Degradation

The photocatalytic degradation effect of SGNPs was evaluated on methyl orange (MO) dye and methylene blue (MB) dye by following the standard method described by [81,82] with slight modification. Concisely, MO and MB (5 ppm) were supplemented with 1.0 mg of SGNPs and kept for incubation at room temperature with light exposure. About 0.1 mL of the test mixture was released at regular time intervals, and its OD value was scanned at 300–900 nm by a spectrophotometer.

### 3.6. Statistical Analysis of the SGNPs

The experimental outcomes are detailed as the mean with SD, and were analyzed via one-way ANOVA and Duncan’s multiple range test at *p* < 0.05 using SPSS statistical software (version 27.0).

## 4. Conclusions

There are many challenges faced by the people of the world due to the harmful effects of different types of microbial infections and other related diseases concerning pathogenic microbes. Despite the availability of several advanced techniques and medications, it sometimes becomes difficult to tackle these pathogenic bacteria due to their multiresistant nature. The unique features of the nanomaterials make them an ideal candidate to apply in treatment against these multiresistant pathogenic microbes and eradicate them. The small particle sizes and shapes of the nanomaterials, along with their charged surfaces, make the nanoparticles an ideal path by which to enter the cell membrane of the pathogenic bacteria, thereby causing cellular lysis in many ways, including the rupture of the cell membrane and destruction of the DNA and protein, and also initiating apoptosis. Additionally, nano-based antibiotic treatment is now being considered as one of the most advanced systematic approaches to tackle harmful microbial pathogenicity. In the present research, the SGNPs exhibited promising antibacterial activity, along with the tyrosinase inhibition photocatalytic degradation potentials. As a consequence of their multifunctional perspective, they could be explored for their future applications as an antibacterial agent, in cosmetics trades as sunscreen, and in the food sector as a coating/film material in the packaging of food, owing to its antibacterial and tyrosinase inhibitory abilities. Furthermore, it can be used in wastewater contamination management of textile dyes. However, a more detailed investigation of the mode of antibacterial action is needed under various conditions and against more pathogenic microbes, including fungi and viruses. Next, detailed studies on the application of the SGNPs in multiple dye degradation scenarios and their kinetics are needed for their potential future applications in the waste toxic dye treatment analysis.

## Figures and Tables

**Table 1:** Antibacterial potential of SGNPs.

Microbes	DZOI *		MIC and MBC Values			
			SGNPs		Cephalexin	
	SGNPs	Cephalexin	IC	BC	IC	BC
**Gram-positive microbe**						
*E. feacium*	8.77 ^b^ ± 0.03	AP				
*S. aureus*	8.67 ^bc^ ± 0.03	ND	50	>50	5.0	>5.0
*L. monocytogenes*	8.61 ^c^ ± 0.02	12.23 ± 1.47	50	>50	5.0	>5.0
**Gram-negative microbe**						
*E. coli*	9.58 ^a^ ± 0.18	AP				
*P. aeruginosa*	8.45 ^d^ ± 0.05	ND	50	>50	5.0	>5.0
*A. hydrophila*	8.54 ^cd^ ± 0.02	12.14 ± 0.41	50	>50	5.0	>5.0

DZOI *—Diameter of the zone of inhibition (measured in mm); SGNPs—sericin-based gold nanoparticles taken at 50 μg/disc; standard antibiotic—Cephalexin taken at 10 μg/disc; IC—minimum inhibitory concentration; BC—minimum bactericidal concentration; MIC and MBC values are in μg/mL concentration; >—greater than; ND—not detected; AP—already published. The data are provided as mean ± standard deviation. The differences in the superscript letters are statistically significant at *p* < 0.05.
